# Imaging of Myocardial Fibrosis in Patients with End-Stage Renal Disease: Current Limitations and Future Possibilities

**DOI:** 10.1155/2017/5453606

**Published:** 2017-03-02

**Authors:** M. P. M. Graham-Brown, A. S. Patel, D. J. Stensel, D. S. March, A.-M. Marsh, J. McAdam, G. P. McCann, J. O. Burton

**Affiliations:** ^1^John Walls Renal Unit, Leicester General Hospital, Leicester, UK; ^2^National Centre for Sport and Exercise Medicine, School of Sport, Exercise and Health Sciences, Loughborough University, Loughborough, UK; ^3^Department of Cardiovascular Science, NIHR Leicester Cardiovascular Biomedical Research Unit, Leicester, UK; ^4^Department of Infection, Immunity and Inflammation, University of Leicester, Leicester, UK

## Abstract

Cardiovascular disease in patients with end-stage renal disease (ESRD) is driven by a different set of processes than in the general population. These processes lead to pathological changes in cardiac structure and function that include the development of left ventricular hypertrophy and left ventricular dilatation and the development of myocardial fibrosis. Reduction in left ventricular hypertrophy has been the established goal of many interventional trials in patients with chronic kidney disease, but a recent systematic review has questioned whether reduction of left ventricular hypertrophy improves cardiovascular mortality as previously thought. The development of novel imaging biomarkers that link to cardiovascular outcomes and that are specific to the disease processes in ESRD is therefore required. Postmortem studies of patients with ESRD on hemodialysis have shown that the extent of myocardial fibrosis is strongly linked to cardiovascular death and accurate imaging of myocardial fibrosis would be an attractive target as an imaging biomarker. In this article we will discuss the current imaging methods available to measure myocardial fibrosis in patients with ESRD, the reliability of the techniques, specific challenges and important limitations in patients with ESRD, and how to further develop the techniques we have so they are sufficiently robust for use in future clinical trials.

## 1. Introduction

Patients with end-stage renal disease (ESRD) are at significantly increased risk of cardiovascular death compared to age-matched peers [1]. The US Renal Data System reports that “cardiac death, cause unknown” and arrhythmia account for 25% of all deaths amongst hemodialysis (HD) patients, at an event rate of 90–200/1000 patient years [2]. These excessive rates of cardiovascular disease (CVD) are unexplained by traditional risk factors [3] and strategies to improve CVD related outcomes, such as coronary artery revascularization, do not improve outcomes for patients on HD [4]. The pathophysiological processes that drive CVD in patients with ESRD are different to those that drive classical atherosclerotic CVD and include chronic inflammation, increased arterial stiffness, autonomic instability, and sympathetic overactivity. These factors lead to the development of changes in cardiac structure and function including left ventricular hypertrophy (LVH), left ventricular (LV) dilatation, diffuse myocardial fibrosis (DMF), and replacement fibrosis (myocardial scarring) [5]. In healthy subjects the extracellular matrix (ECM) makes up around 6% of the normal heart and is composed of hydrated collagen and elastin fibrils produced by cardiac fibroblasts [6]. Its physiological roles include determining tissue mechanics, acting as an anchor for myocytes and as a reservoir for growth factors. In disease states, the volume of ECM can be increased more than 5-fold [7]. A postmortem study of patients with chronic kidney disease (CKD), without coronary artery disease, showed that >90% had myocardial fibrosis (MF) and expanded ECM compartments on histological analysis. Furthermore, levels of MF were more severe in patients on dialysis, progressed over time to replacement fibrosis, and partially regressed in patients who received renal transplantation [8]. This study also suggested that extent and severity of MF were the strongest predictor of death for patients with renal failure.

It has been shown in a number of diseases that increased amounts of MF correlate strongly with the development of arrhythmias and heart failure and sudden cardiac death (SCD) [9–12]; it is likely that this is also the case for patients with renal disease [13]. Additionally MF contributes to diastolic and systolic dysfunction through net accumulation of ECM proteins in the cardiac interstitium that lead to the following: increased LV stiffness; impaired LV diastolic filling; ventricular arrhythmias; and SCD [12, 14, 15]. Three types of MF have been described:

1. Reactive interstitial fibrosis is commonly described in patients with hypertension and diabetes mellitus as a result of activation of the B-adrenergic system and renin-angiotensin-aldosterone system. Stimulated myofibroblasts increase collagen production and deposition in the ECM. It is also present in conditions such as dilated cardiomyopathy and states of pressure overload such as aortic stenosis [16].
2. Infiltrative interstitial fibrosis is less common and characterized by deposition of insoluble substances such as protein tangles in amyloidosis or glycosphingolipids in patients with Fabry's disease.
3. Replacement fibrosis is scarring that occurs after cardiomyocyte damage or once myocardial cell integrity has been affected. The fibrosis may be localised in conditions such as ischemic cardiomyopathy (postmyocardial infarction) affecting a particular coronary territory or a more diffuse distribution of fibrosis as seen in patients with dilated cardiomyopathy [17].

The development of MF in patients with ESRD is complex [18]. Arterial hypertension plays an important role in the pathogenesis of MF in patients with ESRD but is not the only driver. Patients with hypertensive heart disease without CKD develop LVH, diastolic dysfunction and interstitial and perivascular MF related to hemodynamic and humoral factors [14, 19]. In early stage CKD hypertension remains the driving force behind the development of MF, but its influence (whilst still important) reduces as CKD stages progress to ESRD [20]. The importance of the unique environments of CKD and ESRD must not be underplayed in the pathogenesis of MF as the prevalence of LVH and diastolic dysfunction and levels of MF are significantly higher in hypertensive patients with CKD than in hypertensive patients without CKD [21–23]. Arterial stiffness, activation of renin-angiotensin aldosterone system, intravascular volume expansion, oxidative stress, systemic inflammation, and anaemia all contribute to myocardial cell hypertrophy and LV remodelling in patients with CKD and ESRD in addition to arterial hypertension. Pathophysiological responses to these changes lead to activation of pathways that increase production of collagen within the ECM leading to intermyocardial cell fibrosis [24].

The development of DMF and its progression to replacement (scar) fibrosis occur as a continuum. Whilst replacement scar is irreversible, DMF is reversible as it occurs earlier in the disease process. Imaging techniques that allow safe, reliable, early detection of MF may improve disease risk stratification, be an important imaging biomarker in clinical research studies, and allow early treatment to prevent/slow progression to replacement fibrosis or even reverse it.

Some strategies that have targeted LVH reduction in dialysis patients have been shown to reduce SCD and CV morbidity and mortality [25]. There is evidence in animal models, humans, and even HD patients that regression of LVH is accompanied by reductions in MF [26–29]. Given the central association between fibrosis, LVH, morbidity, and mortality in HD patients, being able to reliably measure MF is crucial to understanding its potential as both a research and clinical end point. The purpose of this review is to assess the reliability of the imaging techniques currently available to noninvasively assess MF and DMF in ESRD patients. 

*Imaging and Myocardial Fibrosis in Patients with ESRD.* Identification of MF is historically done with endomyocardial biopsy. Endomyocardial biopsy is prone not only to sampling error but also to significant morbidity and mortality associated with the procedure itself [30, 31]. We will review noninvasive measures of fibrosis using echocardiography and cardiac magnetic resonance (CMR) imaging. Whilst some of these techniques characterize and quantify myocardial tissue directly, others use quantitative measures of cardiac function as surrogates of MF. We will not discuss positron emission tomography-computed tomography, as whilst it could theoretically be used to measure MF in patients with ESRD, the technique is yet to be studied in this population. Contrast enhanced cardiac CT may theoretically also be used to evaluate MF in patients with ESRD, but it has never been used for this purpose in this cohort. Cardiac CT has been used to define MF in patients with hypertrophic cardiomyopathy who are unable to undergo CMR scanning due to having implantable cardiac defibrillators [32] and does seem to be able to define areas of MF in a similar way to certain CMR techniques [33]. However, the technique requires the use of iodinated contrast agents which may affect residual renal function through contrast induced nephropathy [34] and delivers a dose of ionizing radiation. It is for these reasons that its use as a screening tool is not widespread in any population and has never been and is unlikely to ever be used in patients with ESRD.

## 2. Echocardiography

### 2.1. Speckle Tracking Echocardiography

**Figure 1:**
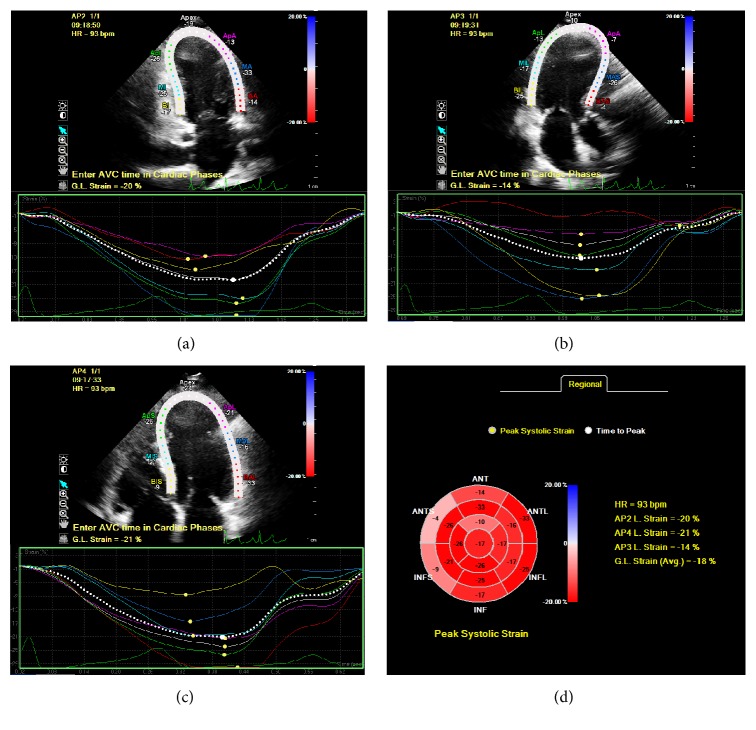
Peak systolic strain assessment using speckle tracking echocardiography. (a) Two-chamber left ventricular strain. (b) Three-chamber left ventricular strain assessment. (c) Four-chamber left ventricular strain. (d) Global and regional strain assessments generated from 2-chamber, 3-chamber, and 4-chamber views.

When imaged by ultrasound, natural acoustic reflections or “speckles” are identified in myocardial tissue (Figure 1). These speckle patterns are unique to the characteristics of each region of myocardium. On imaging, a relatively stable speckle pattern can be identified and defined as a fingerprint in order to track this pattern in the next image frame throughout the cardiac cycle. The speckle pattern can be tracked in two- or three-dimensional images to identify deformation, independent of tethering and translational motion of the whole heart. Speckle tracking echocardiography (STE) was developed in the early 1990s [35] and the use of strain and strain rate imaging has been validated against a number of standards including sonomicrometry [36], tissue Doppler strain measurements [37], and magnetic resonance imaging [38]. Strain describes myocardial deformation and is usually expressed as a percentage change from original point of measurement. During the cardiac cycle, strain represents the lengthening and shortening of the myocardial wall during diastole and systole, respectively. Strain rate imaging represents the rate at which deformation occurs expressed per second. Speckle analysis allows examination of several planes in a single data set (i.e., longitudinal, radial, and circumferential) to define “myocardial strain” [39]. The average longitudinal or circumferential component of strain in the entire myocardium is referred to as global longitudinal strain (GLS) or global circumferential strain (GCS).

The theory behind the relationship between strain, strain rate, and fibrosis is that fibrosis causes hypokinesia of the tissue in affected regions thereby reducing the amount of myocardial wall deformation which results in reduced (less negative) strain values [40], a theory supported by animal studies. Postmortem animal studies by Park et al. demonstrated that radial and longitudinal early diastolic strain rates postmyocardial infarction were significantly related to the extent of interstitial fibrosis measured by the cells-to-collagen ratio (*r* = 0.88 and 0.81, resp.; *p* < 0.01) [41].

Key studies that have used STE in patients with ESRD are shown in Table 1 [42–44]. Kramann et al. induced kidney disease in rats and found that LV strain (peak global radial and circumferential) assessed by STE was significantly associated with myocardial fibrosis on histological examination of rats with kidney disease in comparison to control with correlation coefficients of 0.701 and 0.678, respectively [42]. They extrapolated this information and measured strain in human patients with ESRD and found significantly reduced peak GLS values in ESRD patients in comparison to control (−12.04 ± 3.54 versus −18.37 ± 4.29; *p* < 0.0001). Pirat et al. used STE to compare the strain values of patients on HD, renal transplant recipients, and control subjects matched for age and sex, excluding patients with clinical coronary artery disease. All groups had similar ejection fractions, but average GLS was, again, significantly reduced in HD patients compared to renal transplant recipients, who similarly had impaired GLS in comparison to control subjects (−10.2 ± 1.6% versus −12.5 ± 3.0% versus −14.5 ± 2.9%, resp.; *p* < 0.001) [43]. They also found that average systolic strain rate was similar between controls (−0.76 ± 0.17 per sec) and renal transplant recipients (−0.77 ± 0.21 per sec) and lower in HD patients (−0.62 ± 0.13 per sec; *p* < 0.001) [43]. Chen et al. showed that, despite having normal LV ejection fractions, global three-dimensional strain and regional longitudinal, radial, and circumferential strain were all reduced in the patients with uremia (HD and nondialysis) compared to age, sex, heart rate, and blood pressure-matched controls. They speculated that hypertension, LVH, LV remodelling, and MF all contributed to LV dysfunction in uremic patients with preserved LVEF [44]. These studies suggest GLS may be a subclinical marker of cardiac dysfunction and a way of assessing MF in patients with ESRD, although none of these studies have been validated histologically.

Several studies have looked at STE strain analysis in patients with advanced CKD (not on dialysis) compared to matched HD patients [44–46]. These studies all suggested that strain indices are reduced in patients with advanced CKD compared to matched HD patients. The improvement seen on commencement of HD is almost certainly to do with the improvements in fluid balance and correction of uremia and restoration of acid-base balance that accompany commencement of HD. Levels of MF are known to be greater in HD patients compared to patients with CKD [8, 47], and the observation that strain improves at commencement of HD suggests that measurement of MF with STE in patients with renal failure has many potential confounding factors, including cardiac loading.

A study by Leischik et al. assessed the observer variability of radial, circumferential, and longitudinal strain in 21 healthy controls [48]. Longitudinal strain had the best intraobserver variability (9 ± 13.6% mean deviation, rho = 0.624, and *p* = 0.003), followed by circumferential strain (13.3 ± 8.3%, rho = 0.406, and *p* = 0.068) and lowest in radial strain (26.3 ± 30.1%, rho = 0.391, and *p* = 0.080). Interobserver analyses of longitudinal strain showed best reproducibility (11.9 ± 9.5%, rho = 0.513, and *p* = 0.017), followed by circumferential strain (15.2 ± 12.0%, rho = 0.263, and *p* = 0.249) and the least consistent measurements in radial strain (35.9 ± 46.3%, rho = 0.382, and *p* = 0.088). The use of radial strain should be discouraged due to its poor observer variability. It must also be noted that this study was conducted in healthy control subjects, and the reproducibility of these measures in ESRD patients is likely to be higher, especially given the variability in volume status.

Gayat et al. assessed the agreement of 3D-STE between different vendor software applications in patients with normal LV-systolic function. They found that intertechnique agreement between measures of radial, longitudinal, and circumferential strain was poor with intraclass correlation coefficient < 0.4 [49]. This discordance must, therefore, be taken into account when interpreting 3D deformation data. It should be noted that strain and strain rate can be easily measured with CMR, with superior reproducibility [50], and GCS and GLS have been shown to be significantly reduced in patient with CKD compared to controls (−13.3 ± 2.3% versus −15.9 ± 2.9% and −14.2 ± 1.7% versus −15.9 ± 2.3%, resp.) [51]. Whilst it is possible to measure strain with CMR to assess MF in the same way as with ECHO, no studies in patients with renal disease that have reported CMR-derived strain values have made direct assessments of MF and have tended to report strain as a measure of subclinical systolic and diastolic dysfunction rather than as a tool for assessment of MF.

#### 2.1.1. Integrated Backscatter Analysis

**Figure 2:**
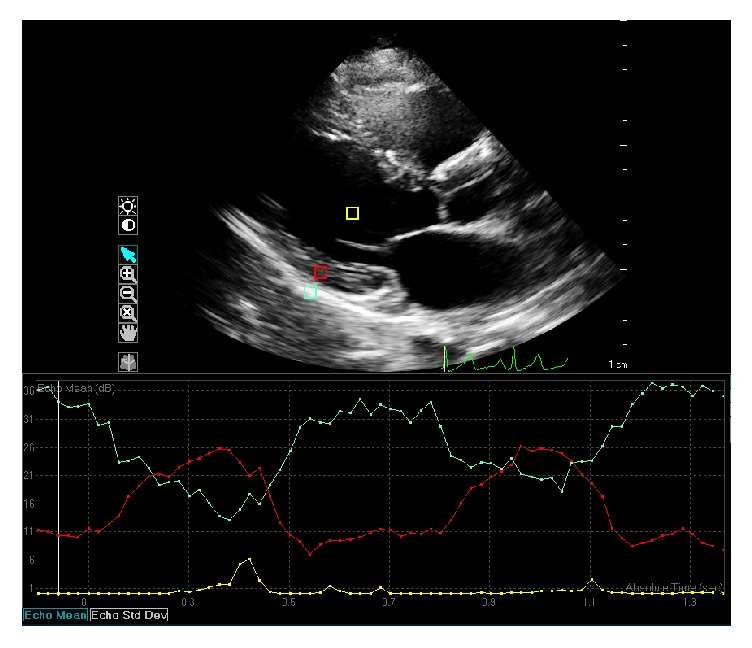
Calibrated integrated backscatter analysis using echocardiography. Region of interest 1 (yellow square) is blood pool within the left ventricle (mean echo-time 0.01 dB). Region of interest 2 (blue square) is the pericardium (mean echo-time 33.96 dB). Region of interest 3 (red square) is myocardium on the posterior wall (mean echo-time 10.29 dB).

Integrated backscatter (IB) is a measure of the ultrasonic reflectivity of a selected part of myocardium, usually the posterior wall. Calibrated integrated backscatter (cIB) uses an intrinsic material such as pericardium (which appears brighter than normal myocardium due to the higher content of fibrosis) or blood (which appears darker) as a frame of reference to compare the reflectivity of myocardium [39] (Figure 2).

Animal studies have demonstrated strong associations between cIB and histologically confirmed levels of MF, with higher IB values associated with the presence of fibrosis [53, 54]. IB has also been correlated with levels of fibrosis in patients with dilated cardiomyopathy [55], congestive heart failure patients [56], and myocardial infarction/ischemic heart disease [57, 58]. Naito et al. demonstrated that cIB was higher in patients with dilated cardiomyopathy than in healthy controls (46.9 ± 6.1 dB versus 42.0 ± 4.4 dB for septum and 37.4 ± 6.5 dB versus 31.0 ± 5.0 dB for LV posterior wall, *p* < 0.01) and that high cIB associated with increased fibrosis in the biopsy specimen of heart tissue (*r* = 0.68, *p* < 0.01 for septum; *r* = 0.71, *p* < 0.01 for LV posterior wall) [55]. Picano et al. studied 16 patients with congestive cardiac failure/suspected cardiomyopathy who underwent LV biopsy and ultrasonic myocardial tissue characterization and found a significant correlation between percent connective tissue area and percent-integrated backscatter index (*r* = 0.55, *p* < 0.05) [56]. However, the authors acknowledged that the myocardial biopsy was not taken from the same area of interest imaged by ultrasound. Not all studies have shown a positive relationship between cIB and histological fibrosis content however. Prior et al. assessed 40 patients undergoing coronary artery bypass grafting and found that levels of fibrosis varied between subjects from 0.7% to 4% and there was no significant relationship between histological evidence of fibrosis on biopsy and preoperative cIB echocardiogram [58]. This study suggested that cIB is less reliable for lower levels of fibrosis. So, cIB may be able to characterize replacement fibrosis but not more subtle diffuse processes. The observer variability of cIB has been assessed in healthy, young subjects, with good intraobserver (*r* = 0.92) and interobserver (*r* = 0.88) reproducibility [59].

Studies that have used IB in HD patients are summarised in Table 2 [60–62]. Salvetti et al. found that mean reflectivity of IB was increased progressively from 48% in patients with essential hypertension to 56% in patients with CKD, further increasing to 62% in HD patients (*p* < 0.01 for all groups) [60]. Although this was not correlated histologically against levels of fibrosis, the authors speculated that increase in IB reflectivity was due to LV collagen deposition, beginning before the development of ESRD. Losi et al. also showed that mean IB was greater in ESRD patients compared to controls (45.2 ± 8.6 versus 36.8 ± 6.1%, *p* = 0.025) and that IB was an independent predictor of diastolic dysfunction (odds ratio = 1.212; *p* = 0.04) [61]. Furthermore, they performed intraobserver variability of IB measurement in 10 consecutive patients, finding variability to be 2%, with a repeatability coefficient of 1.35. A nonrandomised study by Jin et al. assessed the effect of incentre nocturnal HD (INHD) on cIB measurements [62]. They showed that cIB reduced in patients on INHD compared to conventional HD. In addition to a reduction in cIB at twelve months, patients who underwent INHD had a significant reduction in LV mass index, leading the authors to suggest that INHD had reduced levels of MF in the INHD group.

These studies did not assess the correlation between cIB and histological presence of fibrosis in patients with ESRD. Whilst the inter- and intraobserver variability of cIB appear to be good, test-retest reproducibility has not been established and the correlation coefficients described between cIB and histologically proven fibrosis in other disease states are not consistent or as closely matched as necessary for an imaging biomarker.

#### 2.1.2. Common Limitations of Echocardiography

There are limitations to the use of echocardiography for the assessment of MF common to both STE and IB techniques. Measurement of IB and STE by echocardiography is dependent on image quality which varies depending on patient and operator related factors. In the studies of patients with ESRD described above, between 4 and 16.9% of subjects had echocardiographic data excluded due to poor image quality [42, 44, 61]. Strict standardization of methodology is required to reduce the risk of significant intra- and interobserver variability [39, 72], and expertise is required to assure sufficient accuracy and reproducibility of these techniques. Discrepancies between intervendor software analyses mean that studies using different software may not be directly comparable. Finally, echocardiography is inherently dependent on patient volume status and over- or underestimating ejection fraction depending on fluid status [73], a particular problem when dealing with patients with ESRD prone to fluctuations in volume status that undoubtedly affect the reproducibility of STE derived strain values.

### 2.2. Cardiac Magnetic Resonance Imaging

**Figure 3:**
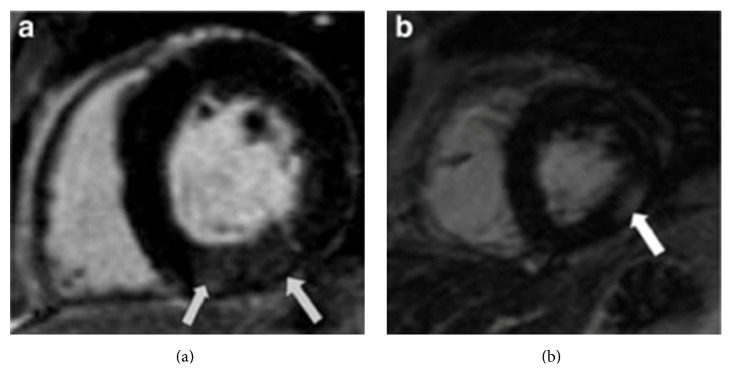
(a) Short-axis view of the left ventricle of hemodialysis patient demonstrating a diffuse area of gadolinium enhancement in the inferior wall of the left ventricle (arrowed). Signal intensity of this area is 17.6 compared to the 6.9 for the LGE-negative area. (b) Short-axis view of the left ventricle of another hemodialysis patient demonstrating a diffuse area of gadolinium enhancement in the lateral wall of the left ventricle. Signal intensity of the area of late gadolinium enhancement is 32.0 compared to 8.4 for the LGE-negative area. This patient had normal coronary arteries at angiography performed as transplant assessment. Image and legend are taken from [52].

CMR is the gold standard in cardiac imaging, with excellent intra- and interobserver variability for assessment of LV volumes and mass [74]. Several CMR techniques may be used to identify myocardial fibrosis. In the general population late gadolinium enhancement (CMR-LGE) using gadolinium based contrast agents (GBAs) is the technique most commonly used for assessing replacement scar fibrosis—often following myocardial infarction. Fibrotic areas show up as bright, due to the increased volume of distribution of gadolinium and its prolonged washout from fibrotic tissue [75, 76] (Figure 3). Mark et al. used CMR-LGE to define the pathological cardiac changes that occur in patients with ESRD [52]. They studied 134 patients with ESRD and performed CMR to assess for LGE. Myocardial fibrosis was found in 28.4% of patients, with two main patterns identified. Subendocardial LGE representing prior myocardial infarction was seen in 14.2% of patients and 14.2% of patients displayed diffuse LGE representing regions of DMF. This latter diffuse LGE was associated with greater LV mass compared to patients without LGE. This is a sensitive and reproducible way of assessing focal MF; however there are limitations in using LGE to assess DMF due to the reliance of the technique on demonstrating a difference between signal intensity of normal and fibrotic myocardial tissue [77]. Extracellular volume (ECV) quantification using pre- and postcontrast T1 mapping sequences (see below) are alternative methods to demonstrate DMF but are also reliant on the use of GBAs to measure the volume of the extracellular space, reflecting interstitial disease and characterizing myocardial tissue [78]. The advantage of this technique over LGE is that it is able to directly quantify the extent of ECM expansion [79].

Unfortunately, CMR-LGE and ECV quantification are not possible in patients with ESRD (or CKD 3–5) due to the risk of Nephrogenic Systemic Fibrosis (NSF) from GBAs [80–82]. As long as GBAs are considered unsafe for use in patients with renal disease, alternative CMR imaging techniques are required to assess myocardial fibrosis in this patient population. Furthermore, given that CMR-LGE has limited ability to define DMF [83], development of novel CMR techniques that can quantify DMF is desirable regardless of the safety profile of GBAs.

#### 2.2.1. Native T1 Mapping

**Figure 4:**
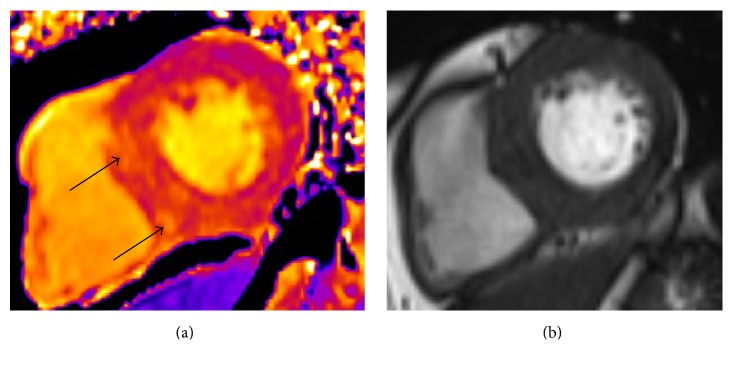
(a) Short-axis midventricular native T1 map of a dialysis patient. Black arrows show areas of discretely increased signal intensity likely to represent myocardial fibrosis. (b) Corresponding short-axis midventricular plain cardiac MRI cine image of the left ventricle of the same dialysis patient. No tissue abnormality visible on plain MR imaging.

Native T1 mapping is a novel noncontrast CMR technique that enhances tissue characterization with CMR. T1 relaxation time is dependent on the molecular environment of water molecules within a given tissue. Native T1 signal can be affected by a number of different factors and T1 mapping characterizes different tissue compositions with great specificity [84]. Myocardial oedema, protein deposition, and interstitial fibrosis will lengthen the T1 recovery time, whilst iron overload and fatty deposits will shorten T1 time (Figure 4).

There are a number of different pulse sequences that can be used to measure myocardial native T1 time, the modified look-locker inversion (MOLLI) recovery sequence and the shortened modified look-locker inversion (shMOLLI) recovery sequence being two examples

Native T1 mapping has been shown to be a useful imaging biomarker in a number of disease states (Table 3). Bull et al. studied 96 patients with severe aortic stenosis (AS) and age-matched controls [63]. They found that T1 values (imaged at 1.5 Teslas) were significantly longer in those with AS than in controls (T1 = 966 ± 41 ms versus 939 ± 19 ms, resp., *p* < 0.001) and there was significant correlation between T1 values and histological fibrosis content (determined by collagen volume fraction) from endomyocardial biopsy (*r* = 0.65, *p* = 0.002) [63]. Similarly Karamitsos et al. compared native T1 times (1.5 T) of patients with AL amyloidosis to those of patients with AS with equivalent degrees of LVH and healthy controls [64]. They found that T1 values were increased in those with AL amyloidosis compared to patients with AS and healthy controls (1140 ± 61 ms versus 979 ± 51 ms, 958 ± 20 ms, both *p* < 0.001). They identified that raised T1 values in amyloidosis patients correlated with systolic and diastolic dysfunction. There was no histological component to this study however, and it is likely that myocardial amyloid protein deposition causes raised native T1 times in these patients. Further work is needed to determine whether there is an absolute native T1 time cut-off or identification of patterns that differentiate amyloid from MF.

#### 2.2.2. T1 Mapping in Patients with ESRD

The potential benefits of T1 mapping in patients with ESRD to define MF are clear [85]. Currently only 3 studies have reported native T1 values in ESRD patients (Table 4). All of these studies were done on a 3-Tesla (3 T) MRI platform and all used the MOLLI sequence to generate native T1 maps.

Rutherford et al. showed that native T1 times in 33 HD patients were significantly higher than 28 age- and sex-matched control subjects imaged at 3 T (1171 ± 27 ms versus 1154 ± 32 ms, *p* = 0.025) [70]. The same study also demonstrated that global native T1 values in HD patients correlated with LV mass indices (*r* = 0.452, *p* = 0.008) and that septal T1 values correlate with predialysis highly sensitive Troponin-T (*r* = 0.397, *p* = 0.027) [70]. These findings were corroborated with findings from our group in a study showing native T1 time are significantly higher in 35 HD patients than 22 comorbidity matched controls (1269.5 ms (1241.7–1289) versus 1085.2 ms (1066–1109.2, *p* < 0.01). Furthermore we found that native T1 times were significantly higher in the interventricular septum of HD patients, compared to nonseptal myocardium (1292.7 ms (1258.9–1310.4) versus 1252.3 (1219.2–1269.6), *p* = 0.002) a difference not present in control patients. Significant correlations were also described between GCS, GLS (measured with CMR), and native T1 values (*r* = 0.41, *p* = 0.002, *r* = 0.55, and *p* < 0.001) [71]. These results are consistent with a study by Edwards et al., which showed significantly longer native T1 times in 129 patients with CKD stages 2–4 (at 1.5 T) compared with age- and gender-matched control subjects and patients with hypertension (986 ± 37 ms versus 955 ± 30 ms versus 956 ± 31 ms, *p* < 0.05) [86]. The study by Edwards et al. also showed a correlation between native T1 time and GLS (*r* = 0.22, *p* < 0.05). The interstudy and interobserver reproducibility of native T1 mapping also appear to be extremely good with coefficients of variation of 0.7% and 0.3%, respectively [87]. Furthermore, early data suggests that changes in fluid status do not affect native T1 signal in HD patients. Our study on the interstudy reproducibility of native T1 mapping showed that whilst changes in weight between scans correlated well with changes in left ventricular end-diastolic volume due to change in volume status and loading, there was no relationship between either change in weight or change in left ventricular end-diastolic volume between scans and change in native T1 time [87].

Wang et al. reported a study of HD patients that whilst native T1 times at 3 T were significantly higher than the established normal range [88] (1273.4 ± 41.7 ms), they were not significantly higher compared to the control group (1253.1 ± 71.6 ms) [89]. The control values quoted by Wang et al. are significantly above the normal ranges for native T1 at 3 T previously published, with no explanation given as to why this might be. The same study did, however, report that native T1-rho values were significantly higher in HD patients than in healthy controls (52.2 ± 4.0 ms versus 49.4  ±  2.6 ms, *p* = 0.001). They also showed the reproducibility of T1-rho mapping was very good, with interobserver intraclass correlation coefficient (ICC) of 0.966, intraobserver ICC of 0.937 for two readers, and test-retest interstudy ICC of 0.836 for the volunteer subjects.

Studies are needed that correlate native T1 mapping with histological specimens from patients with ESRD to demonstrate unequivocally that increased T1 values are due to myocardial fibrosis and not myocardial oedema from fluid overload or any other disease processes.

#### 2.2.3. Limitations of CMR

Whilst CMR is regarded as the gold standard for noninvasive assessment of cardiac structure and function, it is not without limitations. CMR is costly and not widely available and considerable skill is required for image acquisition and analysis. Furthermore postprocessing and analysis of scans can be time-consuming and may require bespoke software applications that are expensive. Additionally there will always be a subset of patients who are unable to undergo CMR scanning due to claustrophobia, metallic implants, or inability to lie flat.

## 3. Conclusions

In 2011, Sado et al. reviewed the imaging techniques to define DMF, including the modalities we have discussed, and laid out a framework for technique development [90]. They concluded that no imaging modality fulfils all the criteria required to confidently define MF and the evidence we have discussed supports this view. To be considered a reliable imaging biomarker of MF, techniques should do the following: be proven to compare closely with histological specimens from human subjects; detect changes in established disease states compared with controls; correlate with cardiac markers of MF (e.g., diastolic function, LVH); correlate with blood biomarkers of MF; be able to track changes over time; and be standardized in the way they are carried out (intervendor/intercentre) and changes in the biomarker should track changes in the disease after treatment.

Echocardiography has limited accuracy and reproducibility in defining geometric parameters and indices of systolic and diastolic function. This is especially true for HD patients who are subject to changes in cardiac filling from fluid status; LV mass and cavity size may be overestimated in up to 50% of dialysis patients [91]. Whilst STE and IB correlate with MF in some diseases, they have not been validated against tissue in HD patients. Furthermore the reproducibility of these techniques appears to lack robustness.

Although modern, more stable, macrocyclic GBAs are now available, which are potentially less toxic [92], it is unlikely they will be approved for use in patients with ESRD. Moreover, as CMR-LGE cannot accurately define DMF [83], development of alternative CMR techniques is needed. Although it is in the early stage of development in patients with ESRD, native T1 mapping shows promise. It correlates well with histology in patients with aortic stenosis [63], is significantly raised in patients with ESRD compared to controls [70, 71], correlates significantly with measures of strain assessed by CMR [71], correlates with circulating markers of cardiac disease [70], and has excellent interobserver and interstudy reproducibility [87]. Further studies are required to correlate native T1 mapping with levels of MF in patients with ESRD and assess the effect of fluid status on T1 values to ensure cardiac oedema is not contributing to the reported raised T1 values. Further data are needed to determine whether T1 is an independent predictor of CV events in patients with ESRD [8].

Whilst imaging biomarkers of MF are attractive measures for future interventional RCTs, we first need more robust and complete data on the relationship between the markers and their relationship to hard outcomes.

## Figures and Tables

**Table 1:** Speckle tracking echocardiography used to assess myocardial fibrosis in patients with ESRD: STE, speckle tracking echocardiography; HD, haemodialysis; PCC, Pearson's correlation coefficient; LV, left ventricular; HR, hazard ratio.

Study	Patient characteristics	Results	Limitations
Kramann et al. [42]	Animal study: 2 rat models with induced kidney disease; clinical study: 171 HD human patients	In rats, peak global radial and circumferential strain was reduced and correlated with interstitial fibrosis (PCC of 0.701 and 0.678, resp.) on histological examination In ESRD patients, mean (±standard deviation) global longitudinal peak systolic was significantly reduced in comparison to healthy subjects (−12.04 ± 3.54 versus −18.37 ± 4.29, resp.; *p* < 0.0001) and this had significant effect on cardiovascular mortality HR = 1.17 (CI: 1.04–1.30; *p* < 0.006)	LV contractility may differ in rats and humans; therefore one cannot entirely extrapolate animal data to dialysis patients

Pirat et al. [43]	Cross-sectional study of 33 patients on HD, 24 renal transplant recipients with functional grafts, 26 age- and sex-matched control subjects	Mean (±standard deviation) global longitudinal systolic strain from the 4-chamber view was highest in control subjects (−14.5% ± 2.9%) and was higher in renal transplant recipients (−12.5% ± 3.0%) than ESRD patients (−10.2% ± 1.6%; *p* ≤ 0.001) demonstrating that myocardial function, quantified by strain imaging, is improved in renal transplant recipients compared with ESRD patients	Cross-sectional data study therefore unable to determine what happens to patients with ESRD after transplant

Chen et al. [44]	Case-control study with 35 HD patients, 30 uremic nondialysis patients, 32 healthy volunteers	LV longitudinal, radial, and global strain values were significantly lower in the nondialysis patients compared with the other two groups (all *p* < 0.001), indicating that reduced strain improves upon starting dialysis. 3D strain and regional longitudinal strain were reduced in HD patients compared to controls (*p* < 0.01)	The nondialysis group had a significantly lower haemoglobin level than the HD and control group. This could be a confounding factor

**Table 2:** Integrated backscatter used to assess myocardial fibrosis in patients with ESRD: IB, integrated backscatter; HD, hemodialysis; CKD, chronic kidney disease; INHD, incentre nocturnal hemodialysis.

Study	Patient characteristics	Results	Limitations
Losi et al. [61]	Case-control study with 25 ESRD patients on HD	Mean (±standard deviation) IB was greater in patients with ESRD than in controls (45.2 ± 8.6 dB versus 36 ± 6.1 dB; *p* = 0.025). A significant determinant of diastolic dysfunction as measured by echocardiography was mean IB with odds ratio of 1.212 (*p* = 0.04)	Small study as very selective patient criteria for inclusion

Salvetti et al. [60]	Case-control study with matched 25 HD patients, 25 patients with moderate-to-severe CKD, 10 patients with essential hypertension with normal renal function	Mean reflectivity of IB was progressively increased from 48% in patients with essential hypertension to 56% in patients with CKD to 62% in HD patients (*p* < 0.01) The increase in IB reflectivity indicates possible early increase in LV collagen deposition, beginning well before the development of ESRD	No histological data from biopsies to confirm fibrosis

Jin et al. [62]	Non-RCT with 58 ESRD patients on conventional HD matched with 32 INHD patients	At 12 months, mean (±standard deviation) cIB decreased significantly from −20.2 ± 3.7 dB to −28.1 ± 4.0 dB (*p* < 0.01) in INHD patients and cyclic variations in IB increased in INHD patients; INHD improved echocardiogram markers for myocardial fibrosis	Nonrandomised, small study with short follow-up time. No histological data from biopsies available to confirm fibrosis

**Table 3:** T1 mapping in myocardial fibrosis caused by different conditions. AS, aortic stenosis; HCM, hypertrophic cardiomyopathy; DCM, dilated cardiomyopathy; LGE, late gadolinium enhancement; LVH, left ventricular hypertrophy; STEMI, ST-elevation myocardial infarction; NSTEMI, non-ST-elevation myocardial infarction; shMOLLI, shortened modified look-locker inversion; MOLLI, modified look-locker inversion.

Condition	Study	Results	Limitations
AS	Bull et al. [63]	Significant correlation between T1 value and histological degree of fibrosis (collagen volume fractions) *r* = 0.65, *p* = 0.002	Due to age matching with patients with AS, older subjects were examined in this study, which could affect the T1 values

Amyloidosis	Karamitsos et al. [64]	Significantly increased mean (±standard deviation) myocardial T1 in patients with AL amyloidosis compared to normal subjects (1140 ± 61 ms versus 958 ± 20 ms: *p* < 0.001). T1 mapping could detect cardiac amyloidosis, even when it was thought to be absent when assessed by echocardiography criteria and standard biomarkers	Results compared to echocardiographic criteria of myocardial amyloidosis and not histological data

Hypertrophic cardiomyopathy and dilated cardiomyopathy	Dass et al. [65]	Mean (±standard deviation) T1 relaxation time per subject was significantly elevated in both HCM and DCM in comparison to controls (HCM 1209 ± 28 ms, DCM 1225 ± 42 ms, control 1178 ± 13 ms, *p* < 0.05). There was modest correlation between T1 mapping and fibrosis identified by LGE; however T1 values were also increased in segments without LGE suggesting distinct pathologies being measured	Small sample size Did not study extracellular volume fractions and therefore can only speculate the mechanisms underlying correlations between T1 values and impaired myocardial function. Used LGE to confirm fibrosis rather than histological analysis

Fabry disease	Pica et al. [66]	Mean (±standard deviation) native T1 in patients with Fabry disease with and without LVH was lower compared to healthy volunteers (853 ± 50 ms and 904 ± 46 ms, resp., versus 968 ± 32 ms, *p* < 0.0001). In patients without LVH, reduced T1 is associated with ECHO parameters of cardiac dysfunction suggesting that a low T1 is detecting early cardiac disease	Small single-centre study. No comparison with biopsy or cardiac magnetic resonance spectroscopy for measuring myocardial lipid storage

Chronic myocardial infarction	Kali et al. [67]	Good agreement between LGE and T1 mapping measuring infarct size (*R*(2) = 0.93 in STEMI and 0.85 in NSTEMI, *p* < 0.05) demonstrating that chronic myocardial infarction size, location, and transmurality can be reliably characterised by T1 mapping	Small sample size and a single-centre study. Did not acquire T2 maps to confirm resolution of acute oedema

Iron overload	Sado et al. [68]	Mean (±standard deviation) myocardial T1 was lower in patients with iron overload than in healthy volunteers (836 ± 138 ms versus 968 ± 32 ms, *p* < 0.0001). T1 reproducibility was also shown to be significantly superior to T2	Significant interstudy and intraobserver differences between the T2 mapping and either of the T1 mapping methods (shMOLLI versus MOLLI)

Acute myocarditis	Ferreira et al. [69]	Using a threshold of T1 > 990 ms (sensitivity 90%, specificity 88%), they found that T1 mapping detected significantly larger areas of myocardial injury (32%) than T2-weighted and LGE (11% and 5%, resp.) imaging in all patients	Differentiation of myocardial areas affected by acute oedema however with no data on chronic scarring/fibrosis

**Table 4:** Studies that have used myocardial native T1 mapping in hemodialysis patients. 3 T, 3-Tesla; MOLLI, modified look-locker inversion; HD, hemodialysis; ms, millisecond; GCS, global circumferential strain; GLS, global longitudinal strain.

Study	Imaging Platform and T1 mapping sequence	Patient characteristics	Results	Limitations
Rutherford et al. [70]	3 T platform MOLLI sequence	33 incident HD patients, 28 age- and sex-matched healthy controls	Mean native T1 values significantly higher in HD patients compared to controls (1171 ± 27 ms versus 1154 ± 32 ms, *p* = 0.025). Native T1 correlated LV mass index (*r* = 0.452, *p* = 0.008) and septal T1 values correlated with predialysis highly sensitive Troponin-T (*r* = 0.397, *p* = 0.027)	No tissue correlation Healthy control patients

Graham-Brown et al. [71]	3 T platform MOLLI sequence	35 HD patients, 22 comorbid matched controls	Median (interquartile range) native T1 times were significantly higher in HD patients compared to controls (1269.51 ms (1241.72–1289.01) versus 1085.2 ms (1066–1109.2, *p* < 0.01). Native T1 times were significantly higher in the interventricular septum of HD patients, compared to nonseptal myocardium (1292.7 ms (1258.9–1310.4) versus 1252.3 (1219.2–1269.6), *p* = 0.002). Significant correlations between GCS, GLS, and native T1 values (*r* = 0.41, *p* = 0.002, *r* = 0.55, and *p* < 0.001)	No tissue correlation No circulating biomarkers of cardiac disease or fibrosis

Wang et al.	3 T platform MOLLI sequence	32 HD patients 35 healthy volunteers	Mean (±standard deviation) native T1 values significantly above the normal range for imaging at 3 T (1273.4 ± 41.7 ms), but not significantly higher than control patients within this study (1253.1 ± 71.6 ms) *p* = 0.157	Control group native T1 values significantly above the normal range. No tissue correlation
